# Mepolizumab asthma treatment failure due to refractory airway eosinophilia, which responded to benralizumab

**DOI:** 10.1002/rcr2.742

**Published:** 2021-04-01

**Authors:** Alistair Cook, John Harrington, Jodie L. Simpson, Peter Wark

**Affiliations:** ^1^ Centre for Healthy Lungs Hunter Medical Research Institute New Lambton NSW Australia; ^2^ Department of Respiratory Medicine John Hunter Hospital Newcastle NSW Australia

**Keywords:** Asthma, benralizumab, eosinophil, mepolizumab

## Abstract

Monoclonal antibodies directed against interleukin (IL)‐5, such as mepolizumab and benralizumab, are an effective and established treatment for severe eosinophilic asthma. Here, we present a patient with eosinophilic asthma with a partial clinical response to mepolizumab initially, as measured by these biomarkers, who when investigated was found to have refractory airway eosinophilia. Escalation of the mepolizumab dose led to further but still only partial response. A treatment trial with benralizumab was more successful and led to suppression of airway eosinophilia. We review the literature, focusing on eosinophil biology at the tissues and the different mechanisms of action of the two agents.

## Graphical abstract

Monoclonal antibodies directed against interleukin (IL)‐5, such as mepolizumab and benralizumab, are an effective and established treatment for severe eosinophilic asthma. Here, we present a patient with eosinophilic asthma with a partial clinical response to mepolizumab, although with refractory airway eosinophilia, who did not fully respond to dose escalation. A treatment trial with benralizumab was more successful and led to suppression of airway eosinophilia.

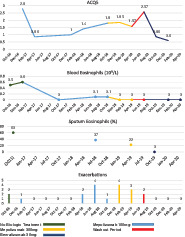

## Introduction

Monoclonal antibodies directed against interleukin (IL)‐5, such as mepolizumab and benralizumab, are an effective and established treatment for severe eosinophilic asthma. Response to therapy is assessed with measurable clinical and biological parameters that have been translated from research, including symptom control scores, use of oral corticosteroids, and suppression of blood eosinophils.

We describe a patient with severe eosinophilic asthma with refractory airway eosinophilia in response to mepolizumab, who demonstrated a clinical and biological response to benralizumab.

## Case Report

A 68‐year‐old man was managed in the severe asthma clinic with adult‐onset eosinophilic asthma and poor disease control despite maximal preventer therapy which necessitated workup and initiation of biological therapy.

The initial onset of symptoms was eight years earlier, and the diagnosis of asthma was made at this time. He had no prior history of asthma or allergy, but chronic rhinosinusitis (CRS) with no prominent occupational or environmental triggers. He experienced one to two severe exacerbations per year that required oral corticosteroids since the initial onset of disease. Daily symptoms included episodic breathlessness, wheeze, and dry cough. The symptoms did not correlate with the work environment, which the patient ultimately retired from due to disease‐related exercise limitation.

CRS was controlled with intranasal budesonide and the patient had previously required a surgical polypectomy. He took no other medications other than asthma therapy. He had a 9 pack‐year smoking history which he had quit 40 years prior. Other potential contributing comorbidities, including gastroesophageal reflux disease (GORD), obstructive sleep apnoea (OSA), and vocal cord dysfunction (VCD), were screened for and ruled out. His body mass index (BMI) was in the normal range (25).

Spirometry demonstrated severe obstruction (pre‐bronchodilator forced expiratory volume in 1 sec (FEV_1_): 1.58 L, 47% predicted) with significant (490 mL; 31%) bronchodilator reversibility. Eosinophilic inflammation was confirmed by the presence of elevated blood eosinophils (0.6 × 10^9^/L). There was elevated fractional exhalation of nitric oxide (FeNO) of 72 ppb. RAST testing demonstrated no IgE response to common aeroallergens. Aspergillus serum IgG was not raised. Anti nuclear antibody (ANA), extractable nucelar antibody (ENA), and anti‐neutrophil cyoplasmic antibody (ANCA) were all negative, as was strongyloides serology (there was no history of travel to tropical areas and the risk was considered low, although the test was performed because anti‐IL‐5 therapy was considered as a possible future treatment option at this point, in order to reduce the risk of disseminated helminth infection). A chest radiograph and computed tomography (CT) were unremarkable—there was evidence of gas trapping on expiratory views and there was no bronchiectasis.

The patient's preventer therapy had been progressively stepped‐up to a high‐dose inhaled corticosteroid (ICS)/long‐acting beta‐2 agonist (LABA) combination (fluticasone/formoterol 250/10 two puffs twice daily) plus add‐on ICS therapy (ciclesonide 160 mcg two puffs daily), although symptoms and blood eosinophils remained elevated despite this regimen. He demonstrated appropriate inhaler technique and reported compliance with therapy. Regular oral prednisolone was initiated, although the patient was ultimately unable to wean below 8 mg per day, and this was still associated with suboptimal symptomatic control (Asthma Control Questionnaire 5 (ACQ5) score consistently >1.5).

The patient was commenced on mepolizumab at a dose of 100 mg subcutaneously. There was a transient improvement of symptoms, and the patient was able to wean off regular oral prednisolone, although suboptimal symptom control (ACQ5 scores consistently 1.0–1.8) persisted and seven exacerbations requiring oral corticosteroids for at least three days occurred over a 12‐month period. Peripheral blood eosinophils were supressed (0–0.1 × 10^9^/L on serial measurements) within weeks of starting treatment. Spirometry was unchanged.

Despite the low blood eosinophils, FeNO remained elevated (53 ppb), suggesting ongoing type 2 airway inflammation. A sputum induction demonstrated refractory airway eosinophilic inflammation despite suppressed blood eosinophils (37% eosinophils (normal <3%); 11% neutrophils (normal <67%)).

A higher dose of mepolizumab of 300 mg was trialled for a period of six months. This led to no significant improvement in symptom control (ACQ5 score: 1.57–1.85 on serial measurements) or exacerbation frequency (seven exacerbations requiring at least three days of oral corticosteroids). An induced sputum after a period of six months of high‐dose mepolizumab demonstrated refractory eosinophilic airway inflammation (22% eosinophils; 30% neutrophils).

**Figure 1:**
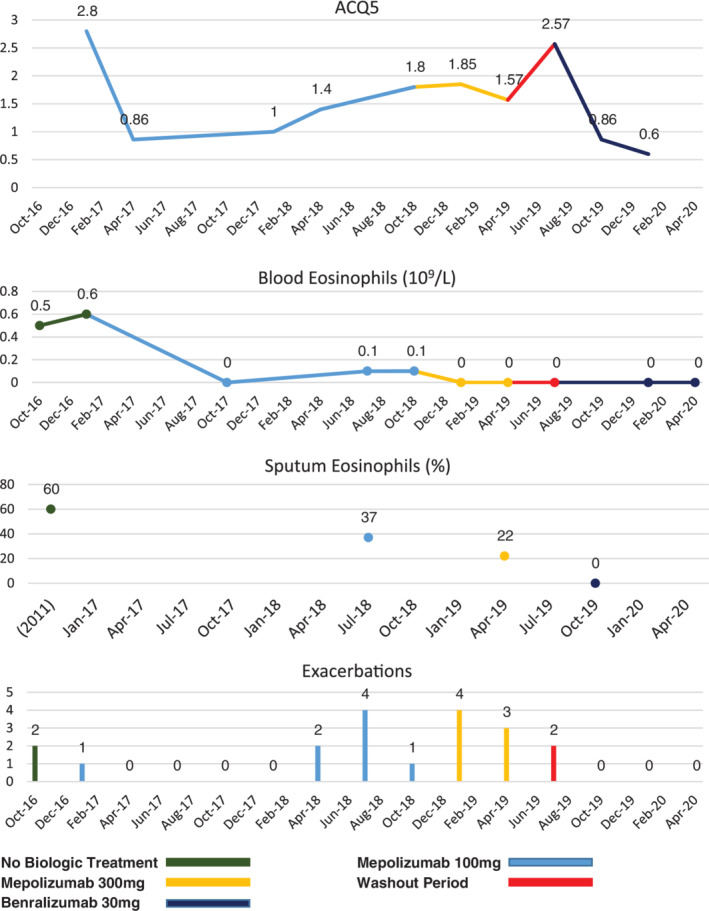
The patient's clinical and biological response to mepolizumab at standard and high doses, and benralizumab over time, as measured by the Asthma Control Questionnaire 5 (ACQ5) symptom control score, frequency of exacerbations, and sputum and blood eosinophils. Exacerbation frequency and symptom control were better while the patient was taking benralizumab compared with either dose of mepolizumab, with a corresponding reduction in sputum eosinophils. Blood eosinophils were supressed while taking both therapies and this did not correlate with clinical response or sputum eosinophils.

Mepolizumab was ultimately ceased, with a view to commence benralizumab via special access scheme. Three months elapsed where the patient was not receiving biological therapy, reflecting the time required to coordinate the application and administer the first dose. During this time, the patient experienced poor symptom control, and two further exacerbations requiring oral corticosteroids, although regular oral corticosteroids were not initiated. After the three‐month wash‐out period, benralizumab was commenced at a dose of 30 mg four weekly. This change to benralizumab was associated with a positive and sustained clinical response, with improved symptoms control (ACQ5: 0.6–0.86 on serial measurements) and no further exacerbations and no further requirement for prednisolone. Peripheral blood eosinophils during this time were consistently undetectable (0.0 × 10^9^/mL). A repeat induced sputum demonstrated suppression of airway eosinophils (eosinophils 0%; neutrophils 48%), indicating suppression of eosinophilic airway inflammation (Fig. 1).

## Discussion

This is a case of severe eosinophilic asthma that is refractory to standard and high‐dose mepolizumab. It did however respond to a greater extent to benralizumab.

Eosinophilic asthma is characterized by a type 2 inflammatory response and the release of the cytokines IL‐4, IL‐5, and IL‐13, with eosinophils as the dominant effector cell [1]. Eosinophilic inflammation has been demonstrated to correlate with the severity of asthma, frequency of exacerbations, and lung function decline [2]. Blood eosinophils are a measurable and generally reliable biomarker that correlates with disease control and treatment effect [1, 3], and clinical response to biological therapy [4], and management strategies that specifically target eosinophilia reduce the risk of exacerbations [5, 6]. Airway eosinophils correlate more closely with disease control than blood eosinophils [7, 8], although they are less accessible in clinical practice.

Persistent airway eosinophilia can promote further type 2 inflammatory cell recruitment and activation at the tissue level. Eosinophils undergo recruitment and activation under the influence of IL‐5, and release mediators that promote inflammation and epithelial cell damage, and modulation of smooth muscle function [9]. Eosinophils are also now known to be the source of the type 2 cytokines IL‐5, IL‐4, and IL‐13 [10], and to have a role in antigen presentation to T‐cells [11], recruitment of dendritic cells [12], and the activation of mast cells [13]. This suggests a larger role in the T2 inflammatory cascade for eosinophils than previously understood.

Patients with eosinophilic asthma, as defined by blood eosinophils >150 × 10^9^/mL, who have severe disease refractory to corticosteroids, benefit from therapy with mepolizumab or benralizumab. Mepolizumab binds to the IL‐5 ligand to inhibit the activation and recruitment of eosinophils, whereas benralizumab binds to the IL‐5 receptor directly. These agents reduce the frequency of asthma exacerbations, reduce corticosteroid dose, and improve symptoms control [4]. There have been no direct head‐to‐head trials comparing these two agents, although their efficacy with regard to these outcomes is similar [4].

The patient was initially administered a mepolizumab dose of 100 mg (the usual dose in clinical practice), although persistent airway eosinophils prompted a trial of a higher 300 mg dose. Phase III trials demonstrated the efficacy of the 100‐mg dose, with a corresponding reduction in blood eosinophils [14, 15]. An earlier trial by Pavord et al. demonstrated an equivalent effect of mepolizumab at doses of 75, 250, and 750 mg with regard to reduction in exacerbation risk [16], although the higher 750‐ and 250‐mg doses resulted in a greater reduction in blood eosinophils than the 75‐mg dose, and only the 750‐mg dose resulted in a statistically significant reduction in sputum eosinophils at 52 weeks [16]. Nair et al. had previously demonstrated that 750 mg mepolizumab can significantly reduce airway eosinophils in patients with prednisolone‐dependent eosinophilic asthma, with a corresponding decrease in exacerbations and corticosteroid dose [17]. Based on the dose–response demonstrated by Pavord et al., a higher 300 mg dose of mepolizumab was used to study its effect in eosinophilic granulomatosis with polyangiitis (EGPA), in which a higher proportion of patients achieved remission with mepolizumab 300 mg compared to placebo [18]. The authors note that although the results are significant, almost half of the patients in this study did not achieve remission, which differs from earlier studies in EGPA in which with higher 750 mg doses of mepolizumab led to remission in 80% of patients [19]. Although the clinical response was not significant, 300 mg of mepolizumab led to a slight reduction in sputum eosinophils in this patient.

A Canadian series similarly described a suboptimal treatment response in 107 of 250 patients with severe eosinophilic asthma treated with mepolizumab or reslizumab [20]. These patients were more likely to have chronic sinusitis and be oral corticosteroid (OCS) dependent. Interestingly, they found them to have elevated IgG antibodies in sputum to anti‐eosinophil peroxidase and increased complement, suggesting the presence of immune complex‐mediated refractory eosinophilic inflammation, without a systemic autoimmune disease [20]. Similarly, they found that blood eosinophils failed to predict clinical response, similar to our case.

Mepolizumab is relatively less effective against airway eosinophils when compared to blood eosinophils [21, 22], although it is not clear if this represents a relatively lack of effect of mepolizumab against IL‐5 in the tissues compared to the bone marrow and circulation. An alternative explanation may be an IL‐5‐independent pathway of eosinophil recruitment and activation in the airways [23]. IL‐5 contributes to eosinophil development, although it may not be as critical to eosinophil survival in the bronchial tissues [24]. Other cytokines such as IL‐3 and granulocyte macrophage stimulating factor (GM‐CSF) have been implicated in the activation and survival of eosinophils in the tissues, especially in the early phase of eosinophilopoiesis [10, 25], and ongoing presence of tissue eosinophils despite anti‐IL‐5 therapy may reflect the effects of these mediators [26].

After being changed from mepolizumab to benralizumab, the patient demonstrated a much better clinical and biological response, reflected in a reduction in airway eosinophilic inflammation. Although this scenario of suppressed peripheral eosinophils and ongoing elevation of airway eosinophils without sustained improvement in response to mepolizumab has been described [27], a subsequent response to benralizumab has not been reported.

The response to benralizumab highlights the different mechanism of action between the two agents. Benralizumab's dual mode of action works by blocking IL‐5‐mediated eosinophil proliferation and survival, as well as enhancing eosinophil apoptosis. Benralizumab is an afucosylated IgG1κ monoclonal antibody and is engineered with enhanced affinity for its binding sites [28]. This modification facilitates antibody‐dependent cell‐mediated cytotoxicity (ADCC), ultimately resulting in a rapid reduction in eosinophils, with near‐complete depletion in bone marrow and tissue [4, 26]. In patients with eosinophilic asthma, benralizumab has been demonstrated to attenuate both airways and blood eosinophils [29, 30] and this is potentially where its advantage lies over mepolizumab.

The advantage of benralizumab may extend beyond a greater reduction in the total numbers of eosinophils. Tissue penetration may be less of a barrier to benralizumab effect, as it is effective even at the low concentrations of its IL‐5Rα target that exist in the tissues, as a function of its ADCC mechanism [28]. By targeting IL‐5Rα, benralizumab also has a broader effect against other cell types that use this receptor such as eosinophils progenitors and basophils [26, 28].

Concerns have been raised about the potential negative effects of such a significant reduction in circulating and tissue eosinophils. Eosinophils are hypothesized to be intrinsically homeostatic cells that regulate local immunity, as well as tissue repair and remodelling, and angiogenesis in both healthy and diseased states [31]. Theoretically, a total reduction of eosinophils could impair these local homeostatic processes, particularly in the thymus, adipose tissue, gastrointestinal (GI) tract, and uterus [24], although current data suggest that a deficiency of eosinophils in animals and humans appears to have no ill effects on normal health [32, 33].

Failure to respond to biologic therapy can occur for any number of pathological, physiological, or extra‐thoracic reasons, in severe asthma and this case underscores the importance of determining the precise mechanism of treatment failure. In this case, refractory airway eosinophilia was driving a suboptimal clinical response, and an escalating strategy to target this, from a higher dose mepolizumab to a change to benralizumab, led to a positive outcome. This case also highlights the relevance of airway eosinophils towards clinical response, and the limitations of relying upon peripheral blood eosinophils to predict a clinical response to anti‐IL‐5 monoclonal antibody therapy.

### Disclosure Statement

Appropriate written informed consent was obtained for publication of this case report and accompanying images.

### Author Contribution Statement

All authors (Alistair Cook, Jodie L. Simpson, John Harrington, and Peter Wark) contributed equally to coordinating relevant clinical information and manuscript preparation.
